# Integrating GC-MS and comparative transcriptome analysis reveals that *TsERF66* promotes the biosynthesis of caryophyllene in *Toona sinensis* tender leaves

**DOI:** 10.3389/fpls.2024.1378418

**Published:** 2024-05-29

**Authors:** Jianhua Dai, Minyan Wang, Hengfu Yin, Xiaojiao Han, Yanru Fan, Yi Wei, Jie Lin, Jun Liu

**Affiliations:** Research Institute of Subtropical Forestry, Chinese Academy of Forestry, Hangzhou, China

**Keywords:** *Toona sinensis*, caryophyllene, AP2/ERF, *Terpene synthase* (TPS), transient overexpression

## Abstract

**Introduction:**

The strong aromatic characteristics of the tender leaves of *Toona sinensis* determine their quality and economic value.

**Methods and results:**

Here, GC-MS analysis revealed that caryophyllene is a key volatile compound in the tender leaves of two different *T. sinensis* varieties, however, the transcriptional mechanisms controlling its gene expression are unknown. Comparative transcriptome analysis revealed significant enrichment of terpenoid synthesis pathway genes, suggesting that the regulation of terpenoid synthesis-related gene expression is an important factor leading to differences in aroma between the two varieties. Further analysis of expression levels and genetic evolution revealed that *TsTPS18* is a caryophyllene synthase, which was confirmed by transient overexpression in *T. sinensis* and *Nicotiana benthamiana* leaves. Furthermore, we screened an AP2/ERF transcriptional factor ERF-IX member, *TsERF66*, for the potential regulation of caryophyllene synthesis. The *TsERF66* had a similar expression trend to that of *TsTPS18* and was highly expressed in high-aroma varieties and tender leaves. Exogenous spraying of MeJA also induced the expression of *TsERF66* and *TsTPS18* and promoted the biosynthesis of caryophyllene. Transient overexpression of *TsERF66* in T. sinensis significantly promoted *TsTPS18* expression and caryophyllene biosynthesis.

**Discussion:**

Our results showed that *TsERF66* promoted the expression of *TsTPS18* and the biosynthesis of caryophyllene in *T. sinensis* leaves, providing a strategy for improving the aroma of tender leaves.

## 1 Introduction

With its long-standing cultivation tradition, *Toona sinensis* is extensively grown as a woody vegetable in the northern and southern regions of China (Peng et al., 2019). Due to the abundance of beneficial secondary metabolites found throughout the plant, it is extensively used in medicine, food production, and the chemical industry (Cao et al., 2019; Zhao et al., 2022). The tender leaves of *T. sinensis* are particularly known for their unique aroma, which contributes to their widespread appreciation. Caryophyllene, propylene sulfide and α-pinene, key raw materials used in producing high-quality chemical products such as essential oils, are common volatile compounds found in the tender leaves of *T. sinensis* (Wang et al., 2023a). Apart from industrial applications, these compounds also show therapeutic potential, making them valuable for treating a variety of diseases. However, significant differences exist in the composition and concentration of volatile compounds found in the tender leaves of *T. sinensis*. In related studies, it was found that significant differences existed in volatile compounds of *T. sinensis* in 8 producing areas (Zhang et al., 2022a). This highlights the significant variations in aroma among different varieties of *T. sinensis*, suggesting the presence of diverse flavour compounds within the species.

Caryophyllene, a sesquiterpene, is a naturally occurring plant compound (MaChado et al., 2018; Francomano et al., 2019; Frank et al., 2021) found in the volatile profiles of various plants, including basil (Sacchetti et al., 2004), black pepper (Geddo et al., 2019; Sudeep et al., 2021) and rosemary (Conde-Hernández et al., 2017). Previous pharmacological studies have demonstrated that caryophyllene possesses a multitude of effects, including the regulation of blood lipids (Baldissera et al., 2017; Youssef et al., 2019), reduction of blood sugar (Basha and Sankaranarayanan, 2016), anti-tumour activity (Ambrož et al., 2015; Dahham et al., 2015) and prevention of liver injury (Cho et al., 2015; Kamikubo et al., 2016). Zhang et al. used GC-MS to investigate and analyse the volatile components of *T. sinensis* from Yunnan Province. This study revealed that the identified volatile components accounted for 92.58% of the total volatile oil. Among these components, caryophyllene has the highest content, reaching 19.51% (Zhao et al., 2022). Wang et al. utilised GC-MS and relative odour activity values (ROAV) to examine the volatile components of *T. sinensis* sourced from the provinces of Guizhou, Yunnan, Sichuan and Shandong. The results indicated that Shandong *T. sinensis* exhibited higher ROAV for caryophyllene compared to those in other regions (Wang et al., 2020). There has been growing interest in the aroma of *T. sinensis* in recent years, as evidenced by an increasing number of studies (Zhai and Granvogl, 2019; Wang et al., 2023). However, existing studies have mainly focused on the collection and classification of germplasm resources (Dai et al., 2023), the extraction of bioactive components (Chen et al., 2009), and the determination and analysis of aroma components (Liu et al., 2013). Genetic improvement of aroma in *T. sinensis* is greatly hindered by a lack of understanding of the molecular mechanisms involved in aroma formation. Caryophyllene is the principal component contributing to the aroma of various *T. sinensis* varieties. A comprehensive understanding of the genetic regulatory mechanism underlying this particular component holds significant potential for breeding novel *T. sinensis* cultivars with desired aroma profiles (Zhao et al., 2017).

Terpene synthase (TPS) is a crucial gene family responsible for forming diverse terpene skeletons (Tholl et al., 2023). It comprises three subfamilies: TPS-c, TPS-e/f, and TPS-h/d/a/b/g (Jia et al., 2022). TPS-c, TPS-e/f are primarily involved in the biosynthesis of ent-kaurene, whereas the TPS-h/d/a/b/g subfamilies are closely associated with secondary metabolism in plants. In *Phoebe bournei*, *PbTPS-a25*, *PbTPS-a21* and *PbTPS-a26* promote the synthesis of β-caryophyllene, and the TPS-a subfamily can promote the synthesis of sesquiterpenes and other terpenoids in plants, enhancing the plant’s resistance to pathogens (Han et al., 2022). Previous studies have indicated that both constitutive and inducible terpenoids play crucial roles in plants’ defence against potential threats. For instance, treatment of Norway spruce with methyl jasmonate (MeJA) induces a complex terpenoid defence response, releasing various monoterpenes and sesquiterpenes (Martin et al., 2004). However, the types and concentrations of terpenoids vary among different plants, correlating with the number and categories of TPS gene clusters within the plants (Tholl and Lee, 2011). The release of terpene volatiles, such as β-ocimene and α-farnesene, in response to insect induction differs among various plant varieties (Huang et al., 2010). Numerous TPS genes involved in terpenoid biosynthesis have been identified in tomatoes, including one encoding an isoprene synthase and seventeen encoding monoterpene and sesquiterpene synthases (Zhou and Pichersky, 2020a). Although many terpenoids are common among plants, some are specific to certain species or groups (Zhou and Pichersky, 2020b). The contents of different terpenoids in the tender leaves of *T. sinensis* indicated that understanding the expression and mechanisms of TPS members is of great significance for further understanding the biosynthesis of terpenoids in *T. sinensis*.

APETALA2/ETHYLENE RESPONSE FACTOR (AP2/ERF) transcription factors play a crucial role in the regulation of terpenoid biosynthesis (Feng et al., 2020), significantly influencing the biosynthesis of various terpenoid compounds in many species such as *Pinus massoniana*, *Catharanthus roseus*, and *Salvia miltiorrhiza* (Paul et al., 2017; Zhang et al., 2019; Zhu et al., 2021). Numerous studies have reported that AP2/ERF transcription factors are essential regulators of hormone signalling, such as jasmonic acid, GA and ABA (Cai et al., 2023; Ma et al., 2024). Under cold stress, the accumulation of terpenoids is promoted by affecting the contents of endogenous hormones, such as jasmonic acid and ABA, and the expression of AP2/ERF family (He et al., 2023). Members of the ERF-IX group respond to jasmonic acid participating in plant secondary metabolism (Imano et al., 2022; Kong et al., 2023). It was discovered that *LcERF19* can bind and activate the promoter of *LcTPS42* to enhance the production of monoterpenoids in *Litsea cubeba* (Wang et al., 2022). Additionally, *CitERF71* was demonstrated to exhibit the capability to interact with the ACCCGCC and GGCGGG motifs within the promoter of e-geraniol synthetase (*CitTPS16*), thereby facilitating being expressed in sweet orange fruits (Li et al., 2017). These studies on ERF transcription factors, especially the ERF-IX group, indicate the critical role in the biosynthesis of terpenoid compounds. However, the regulatory mechanisms of ERF transcription factors in terpenoid biosynthesis in tender *T. sinensis* leaves remain unclear.

The objectives of the study were to identify specific genes associated with terpene biosynthesis through transcriptome analysis, investigate the expression variations among these genes and elucidate their roles in caryophyllene biosynthesis. To accomplish these goals, we analysed the volatile compounds in various parts of the leaves of two *T. sinensis* varieties characterised by distinct aroma compositions. Subsequently, we conducted RNA-seq analysis of these samples to explore their transcriptional variances. We identified the members of the AP2/ERF gene family associated with terpene biosynthesis. Based on the published TPS gene family of *T. sinensis*, we screened members of the AP2/ERF and TPS gene families that may be involved in the biosynthesis of volatile substances, with a particular emphasis on caryophyllene. Additionally, we cloned the target genes and conducted transient overexpression in *T. sinensis* tender leaves to verify the key role in caryophyllene biosynthesis. Our findings highlight the crucial role of caryophyllene biosynthesis in *T. sinensis*.

## 2 Materials and methods

### 2.1 Selection and collection of plant materials

**Figure 1:**
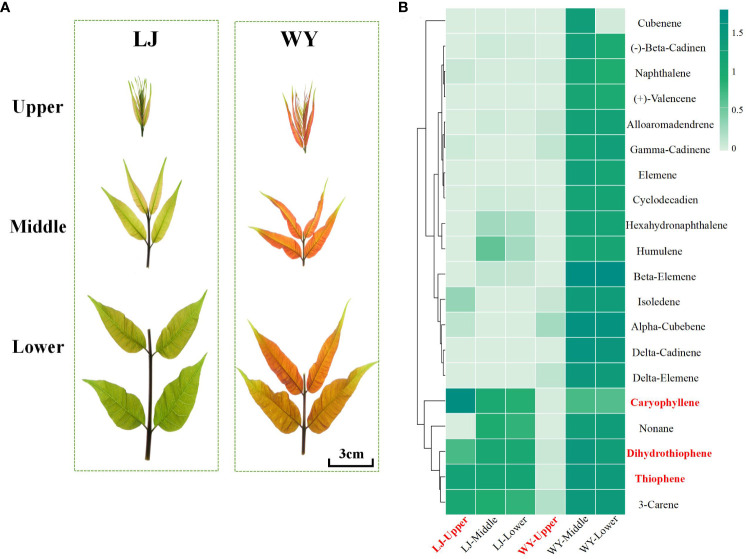
Detection of volatile components in tender leaves of two *T. sinensis* varieties with different aromas. **(A)** Schematic diagram illustrating the sampling strategy for the two *T. sinensis* varieties. Upper, middle and lower represent distinct sections of compound leaves. **(B)** Heat map of the content of the top 20 volatile components.

The *T. sinensis* selected for this study were grown in a nursery on Xinsha Island, Hangzhou, China (Dai et al., 2023). WY variety originated from Sichuan Province, while LJ variety was collected from Shanxi Province. The two *T. sinensis* varieties sourced from different regions exhibit significant phenotypic differences (**Figure 1A**). In this study, we conducted olfactory assessments on *T. sinensis* varieties from multiple geographical areas, selecting LJ and WY based on differences in sensory stimulation of their tender leaves for the determination of volatile substance. We collected the third compound leaf from the top buds of two *T. sinensis* individuals, LJ and WY, at the Xinsha Island nursery in August 2022. The leaves were divided into upper, middle, and lower segments, with three replicates per segment. Subsequently, the samples were rapidly frozen in liquid nitrogen for further experiments.

### 2.2 Detection of volatile substances and RNA-seq

Agilent 8890–5977B (Agilent, California, USA) was utilized to analyse the volatile compound of *T. sinensis* leaves following the Headspace-Solid Phase Micro-extraction Gas Chromatography-Mass Spectrometry (HS-SPME/GC-MS) protocol (Wang et al., 2023b). The specific procedure for determination is as follows: A suitable quantity of these fresh leaves was ground into powder in liquid nitrogen. Next, approximately 0.3 g of the powder was weighed and placed into a sample vial with a lid. Subsequently, 50 μl of a decanoic acid ethyl ester solution with a concentration of 10 μg/ml was added as an internal standard. The heating program settings were shown in **Table 1**.

**Table 1:** Procedure for determination of volatile substances by GC-MS.

Stage	Rate (°C/s)	Temperature (°C)	Hold time (min)	Sample injection port (°C)
1		50	2	250
2	3	80	2	250
3	5	180	1	250
4	10	230	5	250
5	20	250	2	250

Total RNA was extracted using standard methods, and the quantity of RNA were assessed using an Agilent 2100 bioanalyzer. Once the cDNA library passed quality control, Illumina sequencing (NovaSeq 6000) was performed to obtain sequence information by capturing fluorescent signals. Furthermore, we conducted quality control on raw data and calculated the expression matrix (fragments per kilobase million, FPKM) of all unique genes using software such as hisat2, samtools, and featurecounts (Liao et al., 2014; Kim et al., 2019; Zhao et al., 2021). Raw sequencing data were deposited in the NCBI database under the accession number PRJNA1067998,. After obtaining the expression levels of the different genes, we conducted differential expression analysis using DESeq2 to elucidate the expression differences among various samples (Anders and Huber, 2010; Anders et al., 2013; Love et al., 2014). Subsequently, we performed Gene Ontology (GO) (Young et al., 2010) and Kyoto Encyclopedia of Genes and Genomes (KEGG) enrichment analyses (Kanehisa and Goto, 2000) of the differential genes. These analyses enabled us to identify key genes involved in terpene biosynthesis.

### 2.3 Identification of AP2/ERF gene family members

The hidden Markov model (HMM) PF00847 of the AP2/ERF family was downloaded from the PFAM database (Bateman et al., 2004; Zhang et al., 2018). Unique genes were extracted from the *T. sinensis* genome using TBtools (Chen et al., 2020). The HMM search function in TBtools was to identify genes with a conserved AP2 domain. Homologous protein sequences of *Arabidopsis thaliana* (AtAP2/ERF transcription factors) were downloaded from the PlantTFDB 5.0 website (Nakano et al., 2006). Subsequently, the BLAST function in TBtools was used to compare the protein sequences of the AtAP2/ERF transcription factors with those of TsAP2/ERF transcription factors, with e-value less than or equal to 1e-5, identity greater than 45, and bit score greater than 100. Additionally, TsAP2/ERF genes were identified by cross-referencing the results from HMMER and BLAST. The conserved domain search service from NCBI was used for predicting the conserved domains of the candidate TsAP2/ERF genes. Multiple sequence alignment of the TsAP2/ERF sequences in *T. sinensis* was performed using MEGA11 (Tamura et al., 2021), and a neighbour-joining phylogenetic tree was constructed.

### 2.4 Transient overexpression of target gene in *Toona sinensis* and *Nicotiana benthamiana*

*Agrobacterium tumefaciens* GV3101 carries the recombinant overexpression plasmids *TsTPS18* (pNC-Cam1304-MCS35S-*TsTPS18*) and *TsERF66* (pNC-Cam1304-MCS35S-*TsERF66*), along with the pNC-Cam1304-MCS35S empty vector (EV) (Yan et al., 2019), for transient overexpression in *T. sinensis*. The transient overexpression in *T. sinensis* refers to the transient overexpression method in tomato (Wang et al., 2022). *T. sinensis* tissue culture seedlings infected with *A. tumefaciens* were cultured on Murashige and Skoog (MS) medium under a light conditions of 16 hours light and 8 hours dark at 25–27°C for 72 hours. The leaves of *T. sinensis* were collected and stored at –80°C for subsequent analysis, including the determination of volatile compounds and qRT-PCR analysis. Transient overexpression in *N. benthamiana* refers to *A. tumefaciens* infiltration method (Zhang et al., 2022b).

### 2.5 Treatment of *Toona sinensis* leaves with methyl jasmonate

The experimental material for MeJA treatment consisted of two-month-old *T. sinensis* clonal variety grown on MS medium. We added 100 μM L^-1^ MeJA (Merck KGaA, Darmstadt, Germany) to the tissue culture bottles, submerging the plants. The control group was treated with 0.1% ethanol solution (Martin et al., 2002). After 30 minutes of immersion, the solution was poured off, and MeJA and 0.1% ethanol solutions were sprayed separately on the leaves. Each experimental condition was replicated three times at the biological level, with each biological replicate comprising five individual plants. Plants were cultured under full light at 26°C. The tender leaves of *T. sinensis* were collected at 6, 12, and 18 hours post-treatment and stored at −80°C for the determination of volatile substances and qRT-PCR analysis.

### 2.6 Quantitative real-time PCR

The PrimerScript RT reagent kit (Takara, Dalian, China) with gDNA Eraser was used for genomic DNA removal and reverse transcription. The internal reference primer of *T. sinensis* refers to internal reference primer of *Toona ciliata* (Song et al., 2020). The primer information used in this study is provided in **Supplementary Table S1**. QRT-PCR was conducted using the TB Green Premix Ex Taq II kit on ABI/Quantstudio 7 Flex.

## 3 Results

### 3.1 Caryophyllene is a key characteristic volatile compound in *T. sinensis* tender leaves

The tender leaves of *T. sinensis* have a very strong and unique aroma that has attracted attention. Using GC-MS, we detected the volatile components of two cultivars with significant differences in aroma (a strong fragrance termed LJ and a weak fragrance termed WY) (**Figure 1A**). The GC-MS analysis showed that the top 20 components were volatile terpenes and sulfides (**Figure 1B**). Significantly, the main components in the tender leaves were caryophyllene (1.17%–22.23%), dihydrothiophene (0.04%–10.47%), thiophene (0.03%–13.76%), and the content of caryophyllene in LJ (20.89 μg/g) is significantly higher than that in WY (15.37 μg/g). These results suggested that caryophyllene is a key characteristic volatile compound in *T. sinensis* tender leaves, and studying its gene expression and regulatory mechanisms can contribute to the genetic improvement of *T. sinensis* aroma.

### 3.2 Comparative transcriptome analysis in different aroma *T. sinensis* varieties

**Figure 2:**
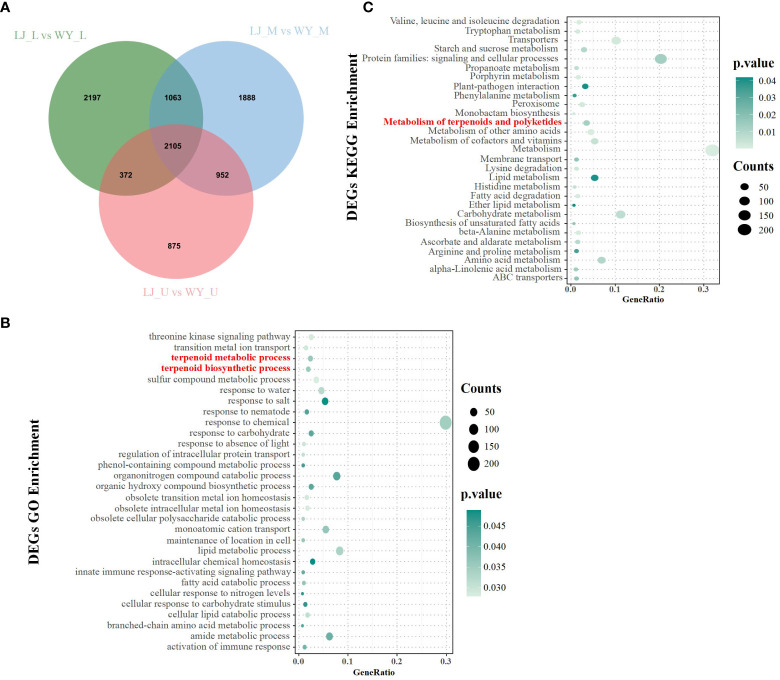
Comparative transcriptome analysis in different aroma *T. sinensis* varieties. **(A)** DEGs in the upper, middle, and lower parts of compound leaves in LJ and WY varieties (*P* < 0.05). **(B)** GO enrichment analysis of all the DEGs. **(C)** KEGG enrichment analysis of all the DEGs. The top 30 enriched GO or KEGG terms are presented. The horizontal axis represents the factors, while the vertical axis represents the GO terms. Counts: number of DEGs.

Transcriptomic analysis was performed to identify the key genes in the leaves of the two cultivars to further explore the mechanism of volatile components enrichment (**Figure 2A**). As a result, comparing the genes in the upper, middle, and lower leaves of the LJ and WY varieties, a total of 9,452 significantly differentially expressed genes (DEGs) were found. Subsequently, GO enrichment analysis showed that the differentially expressed genes in the LJ and WY varieties were significantly enriched in pathways such as responses to chemicals, lipid metabolic processes, terpenoid metabolic processes, and terpenoid biosynthetic processes (**Figure 2B**). KEGG enrichment analysis revealed that the differentially expressed genes in these two varieties were significantly enriched in pathways such as the metabolism of terpenoids and polyketides (**Figure 2C**). These results suggest that the differential expression of genes involved in terpenoid metabolic pathways may explain the differences in the terpenoid compounds in *T. sinensis*.

### 3.3 *TsTPS18* identified as a caryophyllene synthase gene in *T. sinensis* and *N. benthamiana*

**Figure 3:**
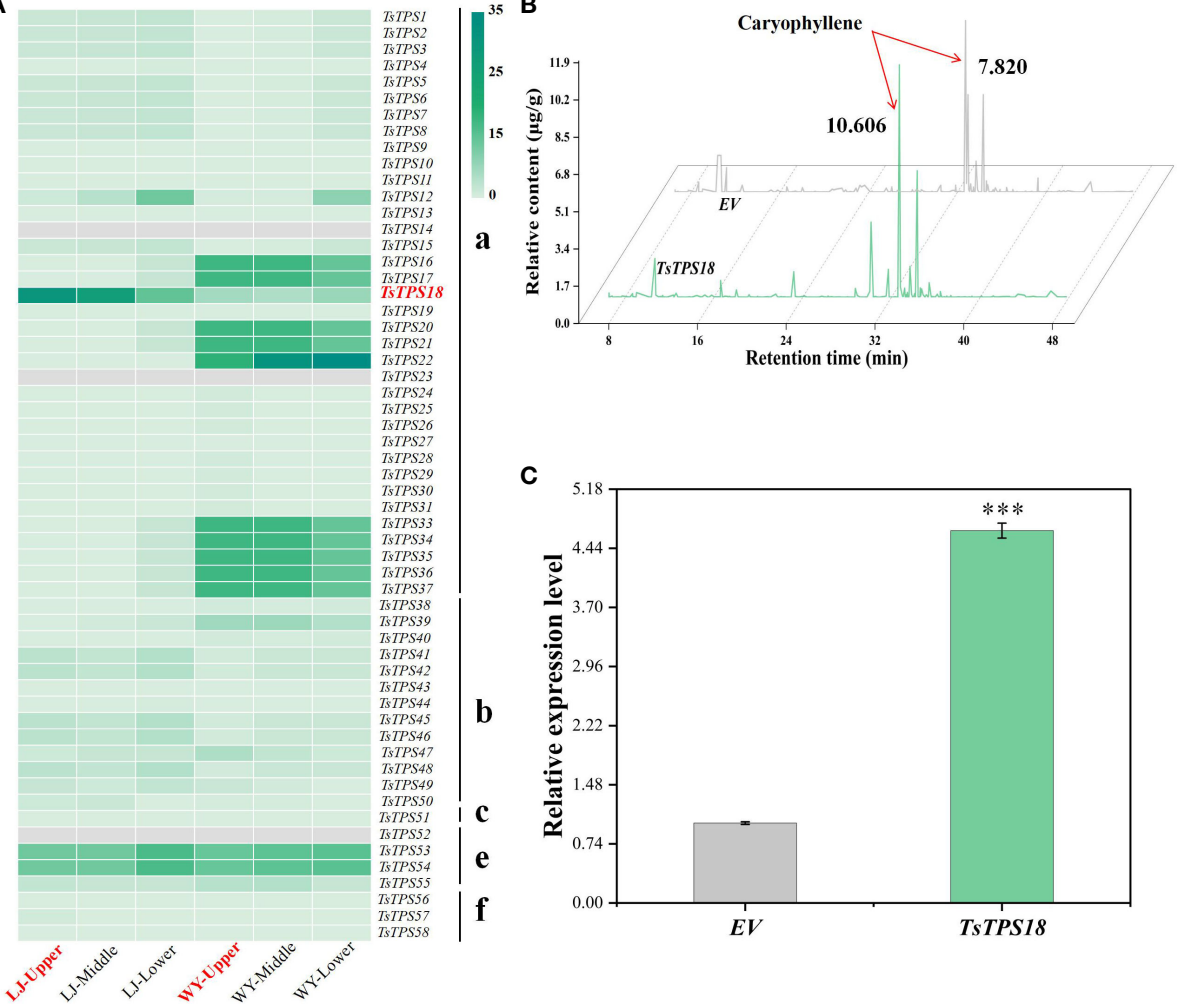
Identification of caryophyllene gene in *T. sinensis*. **(A)** Heat map showing the expression levels of TPS family members in different parts of compound leaves of various *T. sinensis* varieties. **(B)** Detection of volatile components in *T. sinensis* leaves with transient overexpression of *TsTPS18*. **(C)** Relative gene expression in *T. sinensis* leaves with transient overexpression of *TsTPS18*. ***p < 0.001.

**Figure 4:**
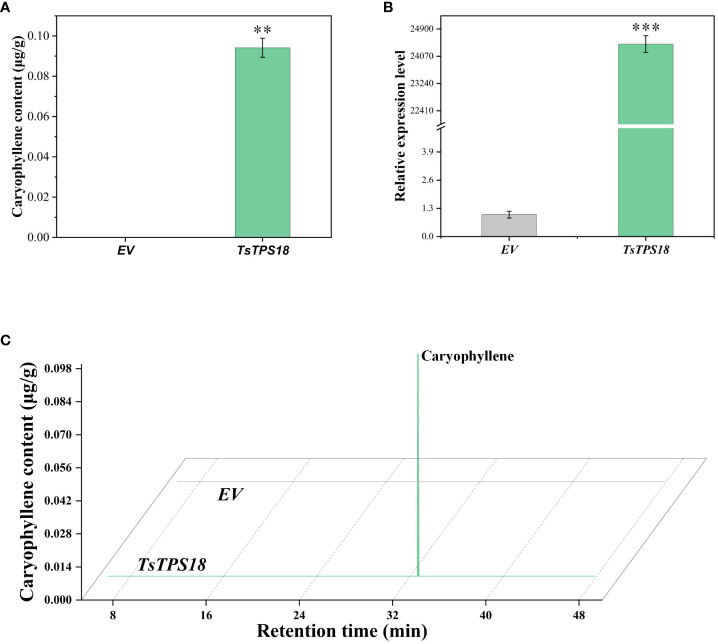
Transient overexpression of the *TsTPS18* gene in tobacco leaves. **(A)** Detection of volatile components in tobacco leaves with transient overexpression of *TsTPS18*. **(B)** Relative gene expression in tobacco leaves with transient overexpression of *TsTPS18*. **(C)** Detection of caryophyllene components in *T. sinensis* leaves with transient overexpression of *TsTPS18*. **p < 0.01, ***p < 0.001.

We investigated the expression of all *TPS* genes as key terpenoids biosynthesis enzymes to further identify the key genes involved in caryophyllene synthesis. Transcriptome expression data showed that the FPKM value of *TsTPS18* in tender leaves was the highest and was significantly higher than that of the other *TPS* genes (**Figure 3A**). Genetic evolutionary analysis suggested that *TsTPS18* is closest to the identified caryophyllene synthase in other plants (Ji et al., 2021) (**Supplementary Figure S1**). In addition, we validated the function of *TsTPS18* by transient overexpression in *T. sinensis* and *N. benthamiana* leaves with a low background caryophyllene content. We injected the leaves of *Agrobacterium tumefaciens* with recombinant *TsTPS18* overexpression plasmids driven by the 35S promoter and an empty vector control (EV), respectively. The results showed that transient overexpression of *TsTPS18* significantly increased the caryophyllene content in *T. sinensis* (**Figures 3B, C**) and *N. benthamiana* leaves (**Figures 4A–C**) compared to that in the control. These results indicated that *TsTPS18* is a caryophyllene synthase gene.

### 3.4 *TsERF66* and *TsTPS18* genes have similar expression patterns and are induced by MeJA

**Figure 5:**
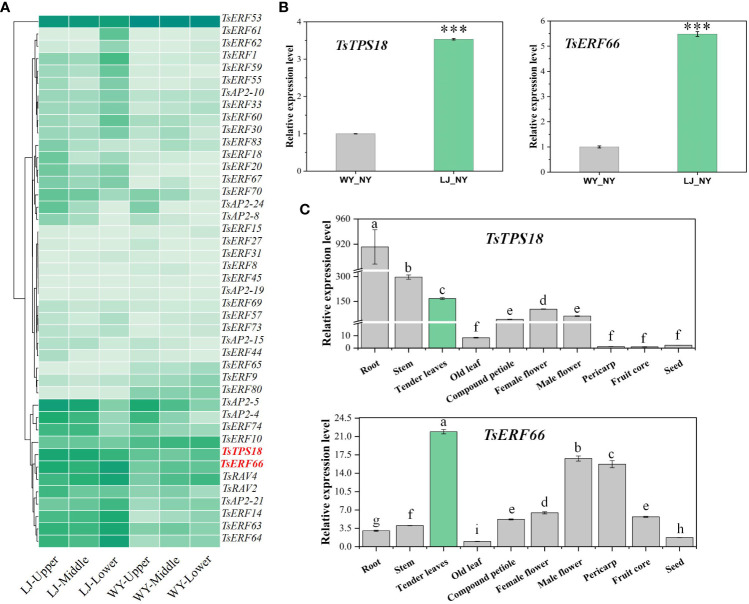
Expression trend analysis of *TsERF66* and *TsTPS18 in T. sinensis*. **(A)** Clustering of expression levels of TsAP2/ERF gene family and *TsTPS18* gene. **(B)** Relative expression analysis of *TsTPS18* and *TsERF66* genes in tender leaves of WY and LJ. **(C)** Relative expression analysis of *TsTPS18* and *TsERF66* genes in different tissues of *T. sinensis*. ***p < 0.001. Symbols a-i represent significant differences between groups.

**Figure 6:**
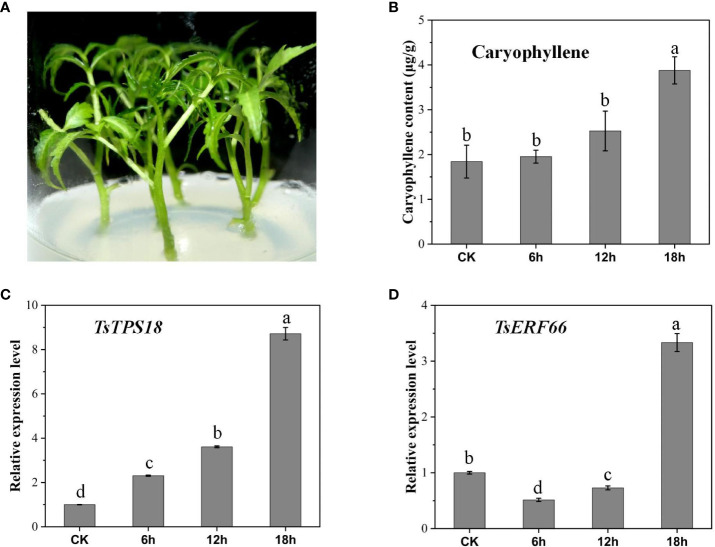
Exogenous MeJA treatment of caryophyllene content and expression of *TsERF66* and *TsTPS18* in *T. sinensis* leaves. **(A)** *T. sinensis* leaves treated with MeJA. The leaves were collected at 0, 6, 9, and 18 h after the treatment. **(B)** Changes in caryophyllene content in *T. sinensis* leaves after MeJA treatment. **(C, D)** Relative expression levels of the *TsTPS18* and *TsERF66* genes in *T. sinensis* leaves after MeJA treatment. Symbols a-d represent significant differences between groups.

AP2/ERF TFs are key in regulating terpenoid biosynthesis, particularly the ERF-IX subfamily, which is a dependent regulatory factor for MeJA that promotes terpenoid gene expression and biosynthesis. In our study, 123 AP2/ERF TFs in *T. sinensis* were identified, classified into subfamilies and named according to *Arabidopsis* classification (**Supplementary Table S3**, **Supplementary Figure S2**). The clustering heatmap showed that the expression levels of the three ERF-IX members were higher in LJ than in WY, and the expression trends of *TsERF66* and *TsTPS18* were consistent with significantly higher expression in tender leaves (**Figures 5A, B**). QRT-PCR showed the same results, and *TsERF66* and *TsTPS18* showed higher expression levels in tender leaves (**Figure 5C**). We further confirmed the relationship between the expression of *TsERF66* and *TsTPS18* and their association with caryophyllene through exogenous MeJA treatment. The results showed that MeJA treatment induced the synthesis of caryophyllene and the expression of *TsERF66* and *TsTPS18* (**Figures 6A–D**).

### 3.5 *TsERF66* promotes *TsTPS18* gene expression and caryophyllene biosynthesis

**Figure 7:**
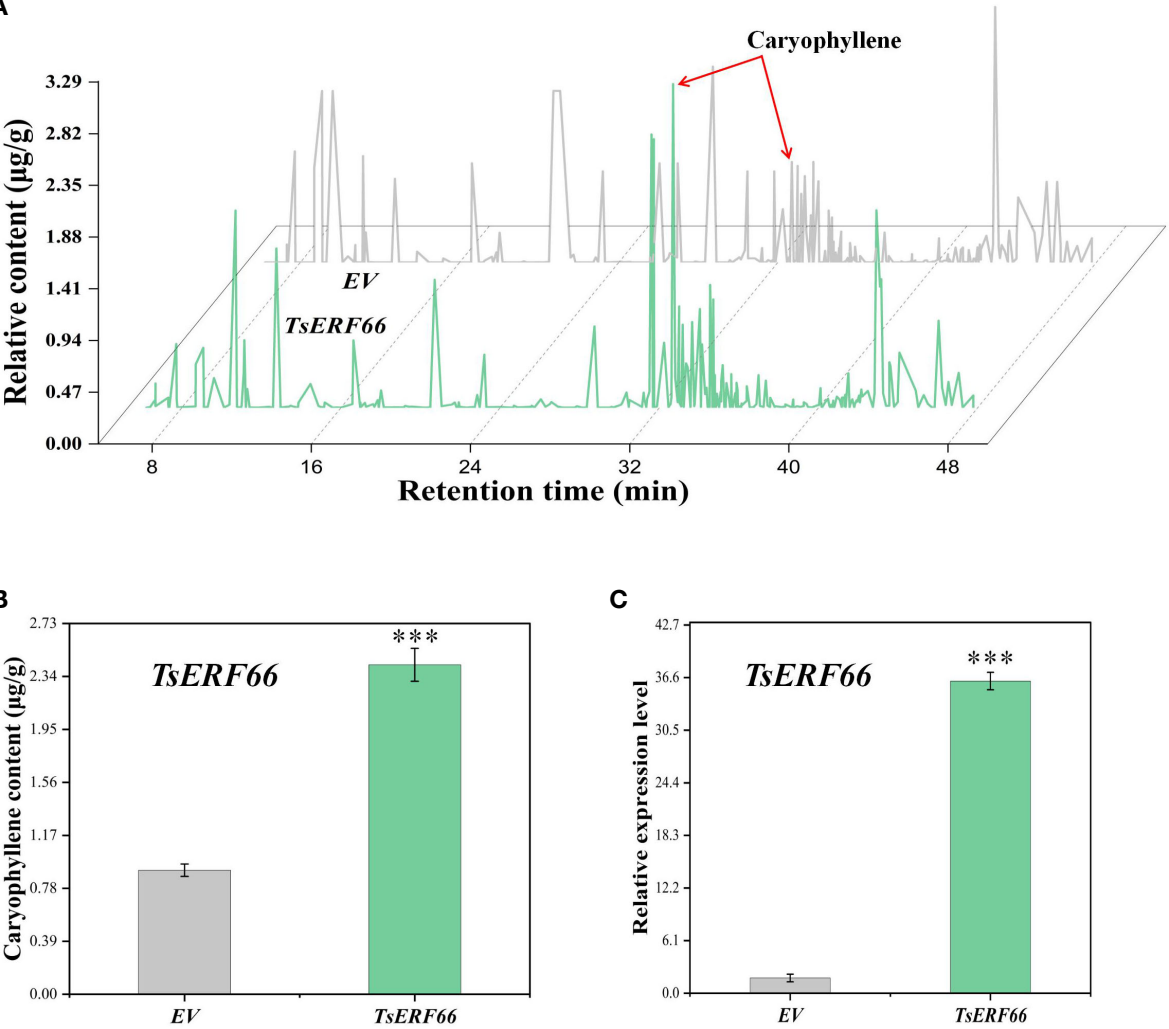
Transient overexpression of *TsERF66* gene in *T. sinensis*. **(A)** Alterations in volatile substances content, as determined using GC-MS, in *T. sinensis* tender leaves after transient overexpression of *TsERF66*. **(B)** Variations in caryophyllene content in tender leaves of *T. sinensis* after transient overexpression of *TsERF66*. **(C)** qRT-PCR results of the *TsERF66* gene in *T. sinensis* tender leaves after transient overexpression of *TsERF66*. ***p < 0.001.

We injected the leaves of *Agrobacterium tumefaciens* with recombinant *TsERF66* overexpression plasmids driven by the 35S promoter and an empty vector control (EV) to confirm the regulatory role of *TsERF66* in caryophyllene biosynthesis in *T. sinensis* leaves. The results showed a 2.5-fold increase in caryophyllene content in *T. sinensis* leaves with transient overexpression of *TsERF66* (**Figures 7A, B**). Additionally, the expression level of *TsERF66* was significantly increased to approximately 36-fold higher than that of EV (**Figure 7C**), suggested that the *TsERF66* assumes a pivotal regulatory role in caryophyllene biosynthesis.

## 4 Discussion

The aromatic profile of *T. sinensis* is a pivotal factor shaping its economic significance. Our analysis of the volatile compounds in the LJ and WY varieties revealed substantial differences in composition and concentration, underscoring the intricate aromatic diversity inherent in these cultivars. The LJ variety, known for its intense aroma, exhibits caryophyllene as the predominant compound, accompanied by sulfur-containing compounds such as thiophene, dimethyl, and dihydrothiophene. However, the main compounds in the less aromatic WY variety were sesquiterpenes, specifically beta-elemene, alpha-cubebene, and delta-cadinene. This result aligned with the findings of other studies on the aroma of *T. sinensis* (Wang et al., 2020). Sesquiterpenes, especially caryophyllene, play a pivotal role in the aroma of *T. sinensis*. To a certain extent, caryophyllene significantly contributes to the sensory evaluation of *T. sinensis*. Furthermore, there were significant differences in the aromatic compositions of the upper, middle, and lower segments of the compound leaves. In the LJ variety, the aromatic composition of the upper segment surpassed that of the middle and lower segments. Conversely, in the WY variety, the aromatic composition of the middle and lower segments significantly exceeded that of the upper segment. This variation might be associated with the developmental status of the plant.

Terpene synthase (TPS) serves as a central enzyme responsible for the biosynthesis of monoterpenes, sesquiterpenes, and diterpenes from precursor molecules such as GPP (geranyl diphosphate), FPP (farnesyl diphosphate), and GGPP (geranylgeranyl pyrophosphat) (Zeng et al., 2016; Zhou and Pichersky, 2020a). Transcription factors, such as AP2/ERF, have been identified in numerous studies as regulators of TPS expression, subsequently influencing the biosynthesis of terpene compounds (Li et al., 2017, 2021; Kumar et al., 2023). In this study, we observed noteworthy enrichment of the sesquiterpenoid biosynthesis pathway among the differentially expressed genes in the LJ and WY varieties of *T. sinensis*, with the *TsTPS18* gene playing a significant role. The expression levels (FPKM) of both *TsERF66* and *TsTPS18* exhibited similar patterns, with higher expression in the LJ variety than in the WY variety. Moreover, qRT-PCR results obtained from tender leaves of both the LJ and WY varieties validated the consistent expression levels of these two genes, which were consistent with the transcriptome results. In *T. sinensis*, transient transformation experiments using overexpression vectors for *TsTPS18* and *TsERF66* further validated their enhanced effects on caryophyllene biosynthesis. Compared with the empty vector control, the overexpression vectors containing either *TsTPS18* or *TsERF66*, resulted in a several-fold increase in the expression levels of *TsTPS18* or *TsERF66* in *T. sinensis*. Additionally, the caryophyllene content increased. The transient transformation experiment in *N. benthamiana* substantiated that the *TsTPS18* gene is the pivotal regulator of the caryophyllene biosynthesis. The involvement of the AP2/ERF and TPS gene families is noteworthy in influencing the aromatic composition of *T. sinensis*, especially in the biosynthesis of sesquiterpenes, such as caryophyllene. Studies have indicated that certain sesquiterpene synthases may produce multiple products (Pazouki and Niinemets, 2016; Tong et al., 2018). Therefore, further investigation is required to understand the regulation of the synthesis of other products by *TsTPS18*. Nevertheless, more in-depth exploration is needed to understand the precise interactions between these two gene families.

Alterations in plant aromatic composition are intricately linked to internal hormone levels and environmental factors (Cheng et al., 2007; Wei et al., 2022; Zhang et al., 2024). MeJA plays a pivotal role in terpenoid biosynthesis in several plant species. However, the precise mechanism through which MeJA regulates caryophyllene biosynthesis remains unclear. Certain studies have proposed that the application of MeJA as an exogenous hormone serves as a non-invasive stressor, provoking plants to activate self-defence mechanisms against external threats. This activation induces the biosynthesis and release of more terpenoids (Martin et al., 2002, 2003; Zulak and Bohlmann, 2010). Moreover, MeJA can activate pertinent transcription factors, including AP2/ERF, MYB, and BHLH (Qi et al., 2018; Huang et al., 2024). MeJA stimulates the upregulation of *AsTPS1* expression through the activation of *AsERF1* in *Aquilaria sinensis*, thereby enhancing agarwood biosynthesis (Li et al., 2021). Notably, several MeJA-responsive cis-acting elements were identified in *TsERF66* and *TsTPS18* genes. We treated the plants with exogenous MeJA to further explore the response mechanism between the *TsTPS18* and *TsERF66* genes and plant hormones. The caryophyllene content exhibited a notable post-treatment increase, accompanied by a gradual upregulation in the expression levels of the two target genes. This consistent trend suggested a potential synergistic interaction between these genes in regulating caryophyllene biosynthesis.

## 5 Conclusion

Caryophyllene, a vital constituent of the volatile compounds in the tender leaves of *T. sinensis*, has significant economic value. We integrated transcriptome and GC-MS volatile substance determination to screen key genes involved in regulating caryophyllene biosynthesis. The regulatory effects of *TsTPS18* and *TsERF66* on caryophyllene biosynthesis were confirmed by gene cloning, transient overexpression, and qRT-PCR in both *T. sinensis* and *N. benthamiana*. Furthermore, we clarified the interaction between them through jasmonic acid-induced treatment of *T. sinensis* and further clarified the biosynthesis mechanism of caryophyllene. This study provides a novel perspective for the genetic enhancement of *T. sinensis* aroma in future studies. Our research revealed the intrinsic mechanism of caryophyllene biosynthesis and regulation in tender leaves of *T. sinensis*. This preliminarily clarified that multiple genes regulate caryophyllene biosynthesis. This study provides crucial theoretical guidance for the development of novel *T. sinensis* varieties with higher caryophyllene production, which is anticipated to accelerate the genetic improvement of superior *T. sinensis* varieties.

## Data availability statement

The original contributions presented in the study are included in the article/**Supplementary Material**, further inquiries can be directed to the corresponding author/s.

## Author contributions

JD: Data curation, Investigation, Validation, Writing – original draft, Writing – review & editing, Visualization. MW: Investigation, Supervision, Writing – review & editing, Visualization. HY: Data curation, Supervision, Writing – review & editing. XH: Supervision, Writing – review & editing. YF: Supervision, Writing – review & editing. YW: Supervision, Writing – review & editing. JLin: Supervision, Writing – review & editing. JLiu: Conceptualization, Funding acquisition, Investigation, Project administration, Resources, Supervision, Writing – review & editing.
