# Supplementary material for: Integrating GC-MS and comparative transcriptome analysis reveals that TsERF66 promotes the biosynthesis of caryophyllene in Toona sinensis tender leaves
Source: Front Plant Sci. 2024 May 29;15:1378418. doi: 10.3389/fpls.2024.1378418 (PMC11171135; doi:10.3389/fpls.2024.1378418)
Supplement: Supplementary file 1 [file DataSheet_1.docx]

**Table S1** Primer details for gene amplification and quantification

| **Class** | **Gene name** | **Primer name** | **Sequence** | **Product size (bp)** |
| --- | --- | --- | --- | --- |
| Amplification primer | TsERF66 | TsERF66-F/R | F: ATGGCATCTCTACAAGAAGCTT  R: TTACATCACCGTCAGTCGAGA | 831 |
| Amplification primer | TsTPS18 | TsTPS18-F/R | F: ATGTCTTCTTCGGTTGCATTA  R: TCATATGGGAATAGGATGGAC | 1689 |
| Quantitative primer | TsERF66 | TsERF66-RT-F/R | F: GAGAGAAAGGTCGCGAAGAA  R: GATAACGGAGGCACACTGAATA | 143 |
| Quantitative primer | TsTPS18 | TsTPS18-RT-F/R | F: AGAGCCTTGCTGTGTTTGTTT  R: TCGTGCTGCATTTTTTTCAT | 228 |
| Amplification primer | Primer adapter |  | F: agtggtctctgtccagtcct  R: ggtctcagcagaccacaagt |  |
| Quantitative primer | Internal reference primer of N. benthamiana | NbActin-RT-F/R | F: TCCTGATGGGCAAGTGATTAC  R: TTGTATGTGGTCTCGTGGATTC |  |

**Table S2** PCR reaction program

| Stage | Pre-degeneration | Denaturation | Annealing | Extension | Final extension | Storage |
| --- | --- | --- | --- | --- | --- | --- |
| Temperature | 95 ℃ | 98 ℃ | 58 ℃ | 72 ℃ | 72 ℃ | 4 ℃ |
| Hold time | 2 min | 10 s | 30 s | 2 min | 7 min | ∞ |
| Repeats | 1 X | 38 X | | | 1 X | 1 X |

**Table S3** Member information of AP2/ERF TFs in *Toona sinensis*

| **Gene names** | **Classification** | **Subfamily** | **New IDs** |
| --- | --- | --- | --- |
| Maker00023307 | AP2 | AP2 | TsAP2-1 |
| Maker00027302 | AP2 | AP2 | TsAP2-2 |
| Maker00033712 | AP2 | AP2 | TsAP2-3 |
| Maker00005544 | AP2 | AP2 | TsAP2-4 |
| Maker00006185 | AP2 | AP2 | TsAP2-5 |
| Maker00026479 | 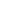AP2 | AP2 | TsAP2-6 |
| Maker00020136 | AP2 | AP2 | TsAP2-7 |
| Maker00016353 | AP2 | AP2 | TsAP2-8 |
| Maker00011893 | AP2 | AP2 | TsAP2-9 |
| Maker00026261 | AP2 | AP2 | TsAP2-10 |
| Maker00016267 | AP2 | AP2 | TsAP2-11 |
| Maker00029381 | 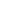AP2 | AP2 | TsAP2-12 |
| Maker00030812 | AP2 | AP2 | TsAP2-13 |
| Maker00009733 | AP2 | AP2 | TsAP2-14 |
| Maker00028349 | AP2 | AP2 | TsAP2-15 |
| Maker00009498 | AP2 | AP2 | TsAP2-16 |
| Maker00034233 | AP2 | AP2 | TsAP2-17 |
| Maker00021634 | AP2 | AP2 | TsAP2-18 |
| Maker00026025 | AP2 | AP2 | TsAP2-19 |
| Maker00033842 | AP2 | AP2 | TsAP2-20 |
| Maker00032881 | AP2 | AP2 | TsAP2-21 |
| Maker00033097 | AP2 | AP2 | TsAP2-22 |
| Maker00025063 | AP2 | AP2 | TsAP2-23 |
| Maker00015911 | AP2 | AP2 | TsAP2-24 |
| Maker00000424 | AP2 | AP2 | TsAP2-25 |
| Maker00032686 | AP2 | AP2 | TsAP2-26 |
| Maker00028561 | AP2 | AP2 | TsAP2-27 |
| Maker00010557 | AP2 | AP2 | TsAP2-28 |
| Maker00025300 | AP2 | AP2 | TsAP2-29 |
| Maker00033006 | AP2 | AP2 | TsAP2-30 |
| Maker00014483 | AP2 | AP2 | TsAP2-31 |
| Maker00014995 | ERF | ERF-III | TsERF1 |
| Maker00015541 | ERF | ERF-III | TsERF2 |
| Maker00000999 | ERF | ERF-III | TsERF3 |
| Maker00022043 | ERF | ERF-III | TsERF4 |
| Maker00029547 | ERF | ERF-III | TsERF5 |
| Maker00025023 | ERF | ERF-III | TsERF6 |
| Maker00007578 | ERF | ERF-III | TsERF7 |
| Maker00029071 | ERF | ERF-III | TsERF8 |
| Maker00023845 | ERF | ERF-III | TsERF9 |
| Maker00028223 | ERF | ERF-III | TsERF10 |
| Maker00004047 | ERF | ERF-III | TsERF11 |
| Maker00008551 | ERF | ERF-III | TsERF12 |
| Maker00004752 | ERF | ERF-III | TsERF13 |
| Maker00019758 | ERF | ERF-III | TsERF14 |
| Maker00006202 | ERF | ERF-IV | TsERF15 |
| Maker00027211 | ERF | ERF-IV | TsERF16 |
| Maker00027073 | ERF | ERF-IV | TsERF17 |
| Maker00009727 | ERF | ERF-II | TsERF18 |
| Maker00028472 | ERF | ERF-II | TsERF19 |
| Maker00004963 | ERF | ERF-II | TsERF20 |
| Maker00015517 | ERF | ERF-II | TsERF21 |
| Maker00015045 | ERF | ERF-II | TsERF22 |
| Maker00005557 | ERF | ERF-II | TsERF23 |
| Maker00005856 | ERF | ERF-II | TsERF24 |
| Maker00026693 | ERF | ERF-II | TsERF25 |
| Maker00020161 | ERF | ERF-II | TsERF26 |
| Maker00017358 | ERF | ERF-III | TsERF27 |
| Maker00023010 | ERF | ERF-III | TsERF28 |
| Maker00014699 | ERF | ERF-III | TsERF29 |
| Maker00015435 | ERF | ERF-III | TsERF30 |
| Maker00001749 | ERF | ERF-I | TsERF31 |
| Maker00012307 | ERF | ERF-I | TsERF32 |
| Maker00017294 | ERF | ERF-I | TsERF33 |
| Maker00002857 | ERF | ERF-I | TsERF34 |
| Maker00020844 | ERF | ERF-I | TsERF35 |
| Maker00012780 | ERF | ERF-I | TsERF36 |
| Maker00033930 | ERF | ERF-I | TsERF37 |
| Maker00018558 | ERF | ERF-I | TsERF38 |
| Maker00007933 | ERF | ERF-I | TsERF39 |
| Maker00021060 | ERF | ERF-VIII | TsERF40 |
| Maker00002051 | ERF | ERF-VIII | TsERF41 |
| Maker00012886 | ERF | ERF-VIII | TsERF42 |
| Maker00021948 | ERF | ERF-VIII | TsERF43 |
| Maker00016710 | ERF | ERF-VIII | TsERF44 |
| Maker00017957 | ERF | ERF-VIII | TsERF45 |
| Maker00012823 | ERF | ERF-VIII | TsERF46 |
| Maker00021062 | ERF | ERF-VIII | TsERF47 |
| Maker00006780 | ERF | ERF-VIII | TsERF48 |
| Maker00005346 | ERF | ERF-VIII | TsERF49 |
| Maker00009001 | ERF | ERF-VIII | TsERF50 |
| Maker00027599 | ERF | ERF-VIII | TsERF51 |
| Maker00005248 | ERF | ERF-VII | TsERF53 |
| Maker00018328 | ERF | ERF-IX | TsERF52 |
| Maker00023803 | ERF | ERF-IX | TsERF54 |
| Maker00019273 | ERF | ERF-IX | TsERF55 |
| Maker00010325 | ERF | ERF-IX | TsERF56 |
| Maker00015173 | ERF | ERF-IX | TsERF57 |
| Maker00027257 | ERF | ERF-IX | TsERF58 |
| Maker00001099 | ERF | ERF-IX | TsERF59 |
| Maker00012649 | ERF | ERF-IX | TsERF60 |
| Maker00012270 | ERF | ERF-IX | TsERF61 |
| Maker00001716 | ERF | ERF-IX | TsERF62 |
| Maker00010271 | ERF | ERF-IX | TsERF63 |
| Maker00031469 | ERF | ERF-IX | TsERF64 |
| Maker00019271 | ERF | ERF-IX | TsERF65 |
| Maker00024832 | ERF | ERF-IX | TsERF66 |
| Maker00014256 | ERF | ERF-X | TsERF67 |
| Maker00003466 | ERF | ERF-X | TsERF68 |
| Maker00022605 | ERF | ERF-VI | TsERF69 |
| Maker00014641 | ERF | ERF-VI | TsERF70 |
| Maker00003543 | ERF | ERF-VI | TsERF71 |
| Maker00005812 | ERF | ERF-VI | TsERF72 |
| Maker00019333 | ERF | ERF-VI | TsERF73 |
| Maker00002796 | ERF | ERF-VI | TsERF74 |
| Maker00004806 | ERF | ERF-VI | TsERF75 |
| Maker00000137 | ERF | ERF-V | TsERF76 |
| Maker00029044 | ERF | ERF-V | TsERF77 |
| Maker00034269 | ERF | ERF-V | TsERF78 |
| Maker00017696 | ERF | ERF-V | TsERF79 |
| Maker00025770 | ERF | ERF-V | TsERF80 |
| Maker00010964 | ERF | ERF-V | TsERF81 |
| Maker00004587 | ERF | ERF-V | TsERF82 |
| Maker00000260 | ERF | ERF-V | TsERF83 |
| Maker00019872 | ERF | ERF-V | TsERF84 |
| Maker00004192 | ERF | ERF-V | TsERF85 |
| Maker00025477 | RAV | RAV | TsRAV1 |
| Maker00015655 | RAV | RAV | TsRAV2 |
| Maker00008167 | RAV | RAV | TsRAV3 |
| Maker00032810 | RAV | RAV | TsRAV4 |
| Maker00034091 | RAV | RAV | TsRAV5 |
| Maker00020081 | RAV | RAV | TsRAV6 |
| Maker00021397 | Soloist | Soloist | TsSoloist |


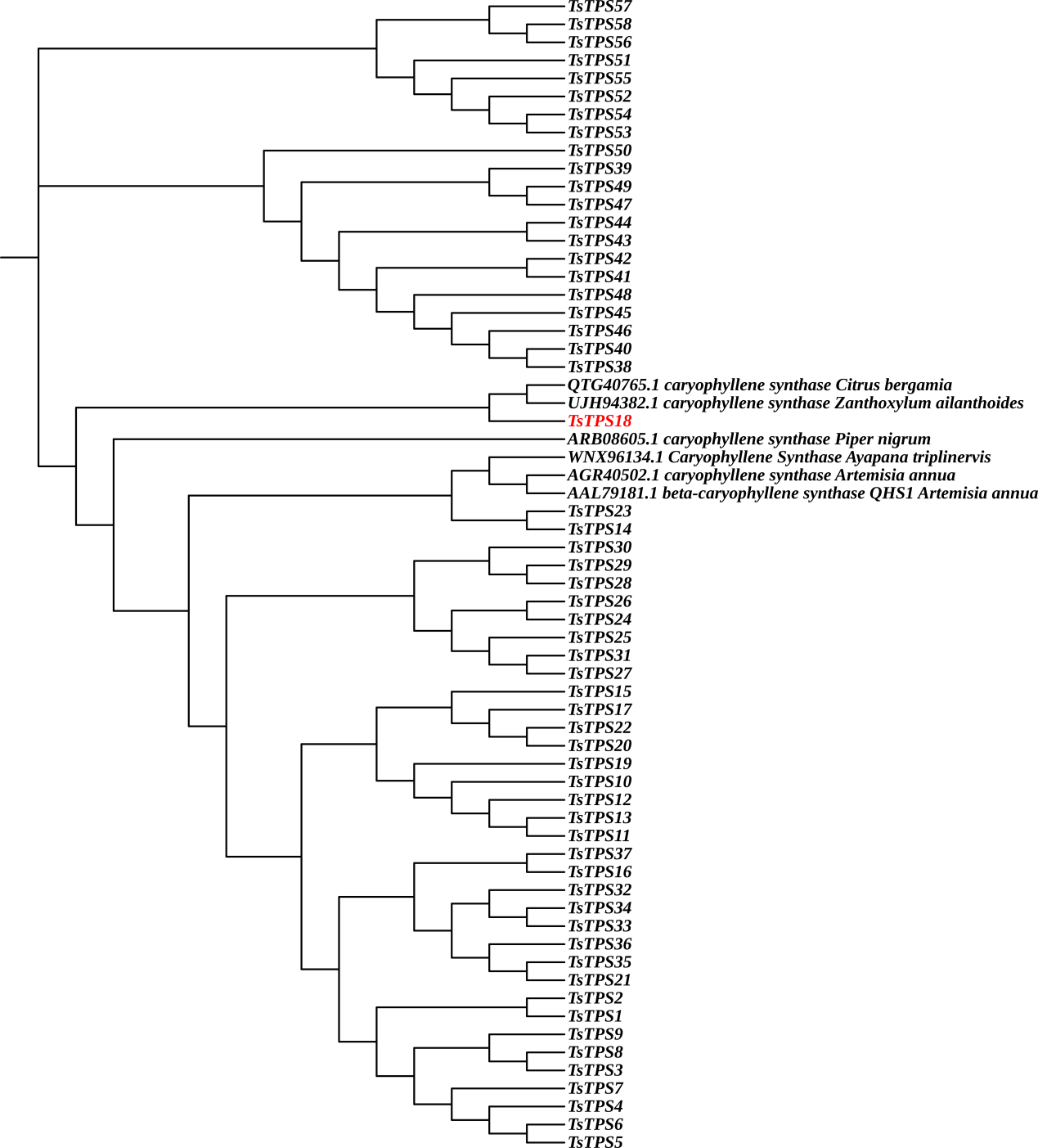


**Fig. S1** Phylogenetic tree of *TsTPS18* gene and caryophyllene synthase genes in other species


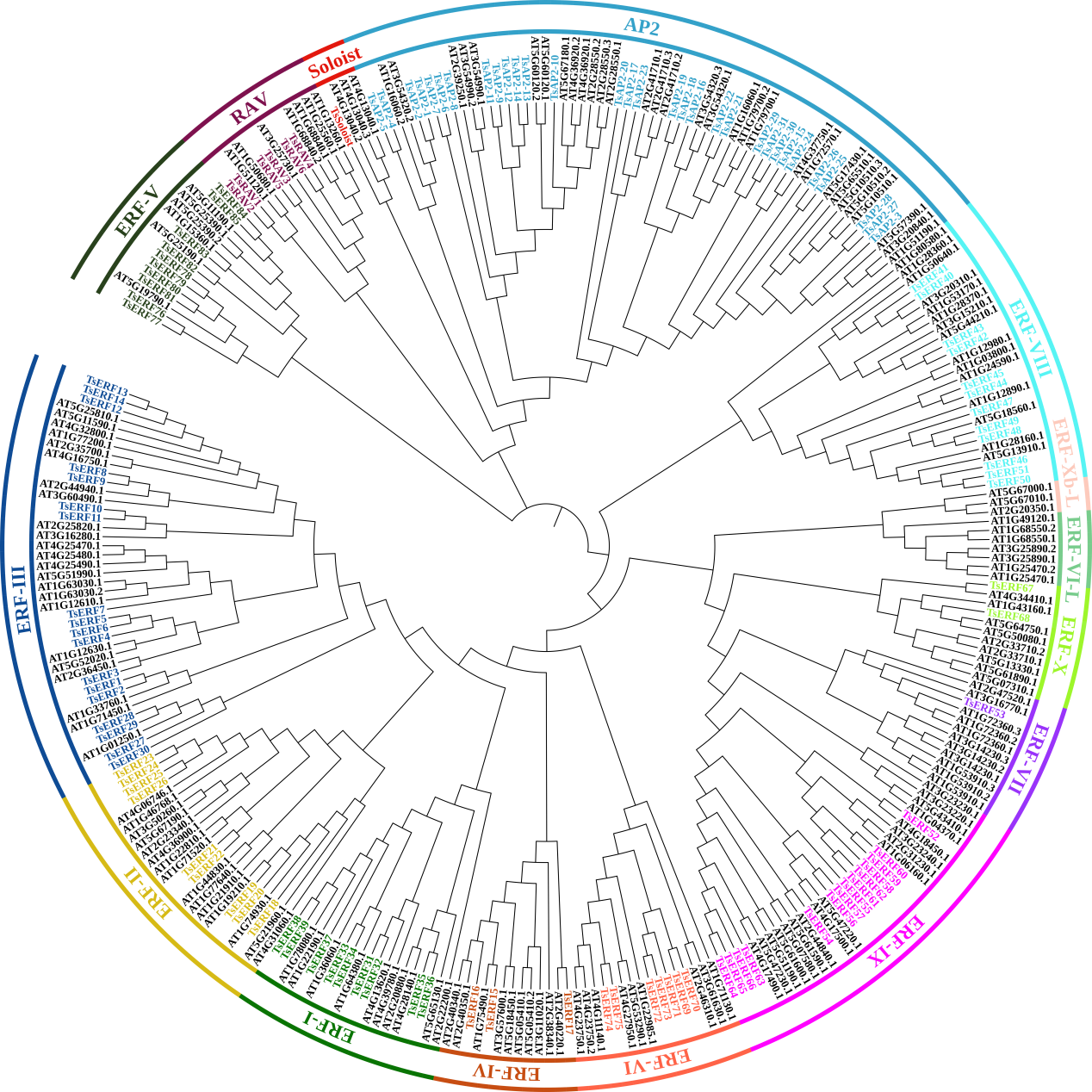


**Fig. S2** Phylogenetic tree of AP2/ERF members of *Toon sinensis* and *Arabidopsis thaliana*
